# Lower Fatigue in the Eccentric than the Concentric Phase of a Bench Press Set Executed with Maximum Velocity to Failure Against Both Heavy and Light Loads

**DOI:** 10.5114/jhk/168792

**Published:** 2023-07-15

**Authors:** Athanasios Tsoukos, Gregory C. Bogdanis

**Affiliations:** 1 School of Physical Education and Sport Science, National & Kapodistrian University of Athens, Athens, Greece.

**Keywords:** velocity loss, electromyographic activity, velocity-based training

## Abstract

We examined changes in barbell velocity and surface electromyographic activity (sEMG) during the concentric (CON) and eccentric (ECC) phases of a bench press set. Ten men executed a set to instant exhaustion as fast as possible, against a low (40% 1-RM) and a heavy load (80% 1-RM), one week apart. The reduction in mean barbell velocity was lower in the ECC compared with the CON phase for both loads (40%1-RM: ECC: −36 ± 21% vs. CON: −63 ± 14%, p < 0.001; 80%1-RM: ECC: −26 ± 15% vs. CON: −59 ± 9%, p < 0.001). Under both loading conditions, sEMG activity of the pectoralis major increased in the last compared to the first repetitions only in the CON phase (by 48.6% and 24.8%, p < 0.01, in the 40% and 80%1-RM, respectively). Similarly, triceps brachii sEMG increased by 15.7% (p = 0.02) and by 21.0% (p < 0.001) during the CON phase in the 40% and 80%1-RM conditions, respectively. However, during the ECC phase, sEMG remained unchanged in the last part of the set for both muscles and loads except for 80%1-RM in the pectoralis major muscle. It was concluded that fatigue measured by velocity loss was lower during the ECC than the CON phase of the bench press movement, when the exercise was performed with maximum velocity to failure, irrespective of the load. sEMG was lower in the ECC than the CON phase for all loads, and increased at the end of the set only during the CON phase, while it remained relatively unchanged in the ECC phase, with the exception of the pectoralis muscle when the load was heavier.

## Introduction

Eccentric (ECC) muscle actions are characterized by different mechanical and surface electromyographic (sEMG) responses and have lower energy requirements compared with concentric (CON) muscle actions (Clarkson and Newham, 1995; Enoka, 1996; Herzog, 2018). Previous research has shown that the force generated in ECC muscle actions is significantly greater than in CON muscle actions under either dynamic or isokinetic conditions (Kelly et al., 2015; Linnamo et al., 2000). Kelly et al. (2015) found that the ECC maximum strength (1-RM) in the bench press exercise was approximately 124% of CON 1-RM in men. Despite the greater force capabilities of ECC muscle actions, sEMG responses have been shown to be lower (Kellis and Baltzopoulos, 1998; Potvin, 1997; van den Tillaar and Sousa, 2020; Westing et al., 1991) or equal (Linnamo et al., 2000) compared with CON muscle actions. For example, Westing and colleagues (1991) showed that EMG activity for all examined muscles was 7% to 31% lower during ECC muscle actions than velocity-matched concentric muscle actions, despite the fact that ECC torque was 20% to 146% higher in all testing angular velocities. This may be due to the engagement of elastic structural elements of skeletal muscles, such as the elastic protein titin, which contributes to the passive force of the muscle-tendon unit by increasing its stiffness (Herzog, 2018). Also, neural drive to the agonist muscles is reduced during eccentric muscle actions, despite maximal voluntary effort (Westing et al., 1991). On the other hand, Linnamo et al. (2000) showed that the average rectified EMG values were the same with the two types of muscle actions despite the higher average force observed under ECC condition. The above conflicting results were obtained by comparing separate ECC and CON protocols. However, there is lack of information regarding changes in sEMG during the CON and ECC phases of the same exercise, and especially the effects of fatigue, when the exercise is performed to failure.

Previous studies conducted on an isokinetic dynamometer, demonstrated that during a fatigue test there was a greater rate of decline in force (−31.6% vs. −23.8%) and sEMG activity (−26.4% vs. −17.5%) when using CON compared with ECC muscle actions (Pasquet et al., 2000). However, other authors found that fatigue resistance was equal in both ECC and CON muscle actions (−25% vs. −26%) (Mullaney et al., 2006). Furthermore, during fatigue (i.e., when performing a set to failure) in a dynamic exercise such as the bench press, barbell velocity during the ECC phase remained unchanged throughout the set, while mean and peak CON velocity decreased by 40% and 28%, respectively (Duffey and Challis, 2007). However, sEMG was not measured in that study, and thus no information exists regarding changes in neural input to the involved muscles under the process of fatigue in the CON and ECC phases of the movement.

In addition to lower fatigue during isolated ECC muscle actions, the number of repetitions to failure in the bench press is higher than isolated CON muscle actions or a combination of ECC and CON muscle actions (Flanagan et al., 2014). Moreover, fatigue resistance and sEMG responses are modulated by loads, with heavy loads exhibiting faster decline in barbell velocity and higher sEMG compared with low loads (Tsoukos et al., 2021b). However, there is no information regarding changes in sEMG and barbell velocity during the ECC and CON phases of a widely used resistance exercise, such as the bench press, against different loads. The ECC phase is defined as the lowering phase during the bench press exercise, that is when the participant lowers the barbell to the chest, whereas the CON phase is the lifting phase when the participant pushes the barbell away from the chest. Thus, the purpose of the present study was to compare barbell velocity loss and sEMG of agonist muscles during the ECC and CON phases of the bench press exercise, executed against different loads (40% of maximum strength 1-RM vs. 80% 1-RM) until failure. We hypothesized that during the ECC phase, fatigue and sEMG would be lower compared with the CON phase and this effect would be observed when using both higher and lower loads.

## Methods

### Participants

Power analysis indicated a minimum sample size of eight participants (effect size: 0.67). This value was taken from a relevant paper on the effects of the bench press exercise to failure on sEMG and mean or peak velocity variables (Tsoukos et al., 2021b). Power analysis was performed using the following variables: the type of analysis was set to repeated measures ANOVA, the required power was set to 0.80, alpha was set to 0.05, and the correlation between repeated measures was set to r = 0.5 (G-Power software, v.3.1.9.2).

Ten strength-trained men participated in the study (age: 27.3 ± 5.9 years, body mass: 79.0 ± 8.4 kg, body height: 1.79 ± 0.07 m, body fat: 12.2 ± 4.2%). Participants were: (a) involved in strength-power sports for over 3 years, (b) free from taking any nutritional supplements or drugs, and (c) injury-free for at least one year prior to the study. Their maximum bench press strength (1-RM) was 99.0 ± 15.6 kg, and their relative strength (per kg body mass) was 1.26 ± 0.18 kg•kg^−1^ body mass. Participants were informed of the potential risks and the right to withdraw from the study at any stage and signed an informed consent form. The Ethics Committee of the School of P.E. and Sport Science of the National & Kapodistrian University of Athens approved the study which was a part of a larger project (approval code/date: 1084/03-10-2018), and the procedures were in accordance with the Code of Ethics of the World Medical Association (Helsinki Declaration of 1964, as revised in 2013).

### Experimental Design and Procedures

This study used a repeated measures experimental design to compare changes in barbell velocity and sEMG activity in the ECC and CON phases in one set of the bench press executed as fast as possible (Salagas et al., 2022) to instant exhaustion against two different loads. Participants took part in two preliminary familiarization sessions and two counterbalanced and randomized experimental conditions (one week apart). The experimental conditions involved a set to failure in the bench press exercise with maximum velocity (tempo of the movement X:0:X:0), against a low (40% of 1-RM) and a heavy load (80% of 1-RM) on a Smith machine. Dependent variables included mean barbell velocity of the ECC and CON phases and sEMG activity [Root Mean Square (RMS)] of the triceps brachii and pectoralis major muscles at the initial (first repetitions) and last (final repetitions) part of the set, recorded during the ECC and CON phases. Since the duration of each repetition was different when performing very fast repetitions during the bench press against lighter and heavier loads, all dependent variables were averaged over the first and last two repetitions for the 80% 1-RM and over the first and last four repetitions for the 40% 1-RM, in order to match time under tension and thus compare the two conditions on an equal time under tension basis (Tsoukos et al., 2021b).

### Baseline Measurements and Familiarization

Participants took part in two preliminary sessions. Anthropometric measurements were taken, and the maximum bench press (1-RM) was evaluated during the first preliminary visit. During the second familiarization visit, participants performed a general warm-up, followed by a specific warm-up. Then, they performed a familiarization set of 20 repetitions against 40% 1-RM and a set of 5 repetitions against 80% 1-RM, with 5 min of recovery in between. All repetitions were executed as fast as possible from the start of the exercise, and these sets served to familiarize the participants with the main test conditions.

### General and Specific Warm-Up

A standardized general warm-up was performed before all preliminary and main sessions. This included five minutes of light exercise on a cycle ergometer (50–60 W) followed by five minutes of dynamic stretching of the upper limb muscles (Tsoukos et al., 2021a). Subsequently, participants performed a specific warm-up which included a set of eight repetitions against 50% of the load that followed (40 or 80% 1-RM). After 3 minutes of rest, participants performed a set of five repetitions against 75% of the load that followed.

### Anthropometric Measurements

A stadiometer was used to measure body height (Charder HM-200P Portstad) and a scale for the assessment of body mass (TBF-300A Body Composition Analyzer-Tanita). A Harpenden skinfold caliper (British Indicators Ltd., Herts, England) was used for the estimation of body fat (Jackson and Pollock, 1985).

### Maximum Bench Press Strength

Maximum bench press strength or one-repetition maximum (1-RM) was evaluated on a Smith machine (Baechle et al., 2008). Participants were tested in the supine position with the head, shoulders and the trunk supported by the bench, the knees bent (90^o^) and the feet rested flat on the floor. The barbell was grasped slightly wider than shoulder-width with a pronated grip. Two experienced strength and conditioning coaches acted as spotters. Participants completed one set of 3–5 repetitions at 50–60% of the predicted 1-RM, one set of 2–3 repetitions at 75–80% of the predicted 1-RM and 3 to 5 sets of one repetition to determine the 1-RM (Baechle et al., 2008). The intraclass correlation coefficient (ICC) was calculated using a two-way mixed-model analysis of variance (ANOVA) and for the measurement of 1-RM it was found to be 0.923 (95% CI: 0.812–0.975, *p* < 0.01).

### Barbell Velocity Measurements

A linear position transducer (Tendo Power Analyzer System v. 314, TENDO Sports Machines, Trencin, Slovak Republic) was used to measure barbell velocity. The device was placed vertically to the movement of the barbell. Mean barbell velocity during the upward (concentric) and downward (eccentric) movement of the barbell was measured for every repetition. The ICC for mean barbell velocity in our laboratory is 0.983 (95%CI: 0.962–0.995) (Tsoukos et al., 2019, 2021a).

### Surface Electromyographic (sEMG) Activity

sEMG activity of prime movers (pars sternocostalis of the pectoralis major and lateral head of the triceps brachii) was recorded throughout the bench press exercise using appropriate instrumentation (Biopac MP35, systems Inc., Santa Barbara, CA) and software (Acqknowledge 4.2.0, Biopac Systems Inc., Santa Barbara, CA). Bipolar electrodes (Ag/AgCl) were placed on the pectoralis major and triceps brachii of the dominant side (Krzysztofik et al., 2021) according to the recommendations of SENIAM (Hermens et al., 2000). The inter-electrode distance was 20 mm, and the ground electrode was attached on the clavicle. Skin over which the electrodes were placed was shaved, cleaned with alcohol and then rubbed with fine sandpaper to maintain low impedance between the electrodes. sEMG data were sampled at 2000 Hz and raw signals were amplified (gain = 1000), bandpass filtered (low-pass cut off frequency: 30 Hz and high-pass cut off frequency: 500 Hz, FIR Blackman 61db) (Girard et al., 2018) and smoothed by the root mean square algorithm with a 100-millisecond sliding window (Kellis et al., 2017; Mausehund et al., 2022). The onset of each burst was determined manually by the same investigator in all sEMG data, and the Root Mean Square (RMS) value was calculated. The RMS values were normalized to the peak RMS obtained from the maximum voluntary isometric contraction (MVIC) performed before each condition (%MVIC) (Besomi et al., 2020). The MVIC determination included two maximum isometric bench press actions with an elbow angle of 90^o^. Each repetition lasted 3 s and the two repetitions were separated by 3 min rest intervals (Paz et al., 2017). This process was performed ten minutes before each experimental protocol. The average of the two peak sEMG values was used for normalizing sEMG during the bench press sets to failure. The ICCs for the measurements of sEMG in our laboratory are: 0.956 (95%CI: 0.904–0.984) for the pectoralis major and 0.933 (95%CI: 0.854–0.976) for the triceps brachii (Tsoukos et al., 2021b). Eccentric and concentric muscle action was determined by an electro-goniometer (Biopac SS21L, Biopac Systems, Goleta, CA) placed on the elbow joint.

### Calculations

Volume load (kg) was determined as the product of the lifted load and the number of repetitions. sEMG and mean barbell velocity data were matched by averaging a specific number of repetitions at the first part of each set (that is 4 and 2 repetitions for 40% and 80%-1-RM conditions, respectively), so that the partial time under tension (pTUT) was the same for the two loads at the initial part (40% 1-RM: 3.06 ± 0.40 s vs. 80% 1-RM: 2.68 ± 0.45 s, *p* = 0.89). This process was carried out because each repetition had different duration when exercising against 40% 1-RM and 80% 1-RM loads. Data were similarly averaged at the end of each set, i.e., the last 2 and 4 repetitions of the set for 80% and 40%-1-RM, respectively. Velocity loss from the initial to the last part of each set was calculated as a percentage decrease in mean barbell velocity, as defined above.

### Statistical Analysis

All measured variables presented a normal distribution (Shapiro-Wilk test) and sphericity was verified (Mauchly’s test of sphericity). Data are presented as means and standard deviations (SD). Changes in sEMG and barbell mean velocity were compared using a 3-way repeated measures analysis of variance (ANOVA) (2 loads [40 and 80% 1-RM] x 2 parts [initial and last part] x 2 muscle actions [ECC and CON]). When appropriate, a follow-up 2-way ANOVA was also conducted. A Tukey’s post-hoc test was performed when a significant main effect or interaction was observed. Differences between conditions in the lifted load (kg), the number of repetitions performed, and the volume load were examined using dependent *t* -tests. Partial eta squared (η2) was used as a measure of effect size in ANOVAs (effect size classification: small: 0.01 to 0.059, moderate: 0.06 to 0.137 and large: >0.137), while Hedges’ *g* was used to calculate effect size for pairwise comparisons (small, <0.30; medium, 0.30–0.80; large, >0.80). Statistical significance was accepted at *p* < 0.05. All analyses were performed using the SPSS Statistical package v. 23 (IBM Corporation, USA).

## Results

### Lifted Load, Total Number of Repetitions, Total Volume Load and Time Under Tension

The lifted load was significantly greater under the 80% 1-RM condition compared to the 40% 1-RM (79.3 ± 12.2 vs. 39.7 ± 6.2 kg, *p* < 0.001, Hedges’ *g* = 3.84). In contrast, the volume load and total number of repetitions were higher in the 40% 1-RM compared with the 80% 1-RM condition (repetitions: 45.6 ± 5.9 vs. 10.2 ± 1.9, *p* < 0.001, Hedges’ *g* = 7.78; volume load: 1807 ± 360 vs. 804 ± 176 kg, *p* < 0.001, Hedges’ *g* = 3.39).

The two-way ANOVA revealed a significant interaction for pTUT (*p* = 0.044; η2 = 0.38). Tukey post-hoc tests showed that pTUT at 40% 1-RM and 80% 1-RM was similar under the two conditions at the initial part (40% 1-RM: 3.06 ± 0.40 s vs. 80% 1-RM: 2.68 ± 0.45 s, *p* = 0.89). However, pTUT was longer in the last part at the 40% 1-RM condition compared with the 80% 1-RM condition (40% 1-RM: 7.01 ± 2.10 s vs. 80% 1-RM: 4.88 ± 1.16 s, *p* = 0.013, Hedges’ *g* = 1.21).

### Mean Concentric and Eccentric Barbell Velocity, and Velocity Loss

**Figure 1:**
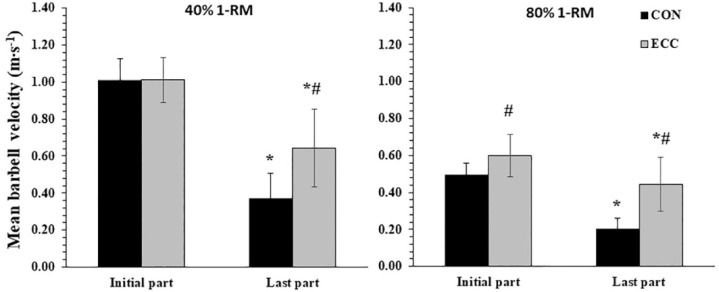
Mean barbell velocity at the initial and the last part of the set during the 40% of 1-RM (mean of 4 repetitions) and 80% of 1-RM conditions (mean of 2 repetitions). *: *p* < 0.01 from the initial part; #: *p* < 0.01 from CON muscle actions in the corresponding load and part

ANOVA showed a significant three-way interaction (load x muscle action x part) for mean barbell velocity (*p* = 0.038; η^2^ = 0.31). Mean ECC and CON velocities were significantly higher in the initial compared with the last part for both conditions (*p* < 0.01, Figure 1). Furthermore, mean barbell velocity was higher at the 40% 1-RM load compared with the 80% 1-RM load in all parts and muscle actions (*p* < 0.01). Also, ECC mean velocity was higher at the initial part and the last part under 80% 1-RM condition compared with the CON mean velocity (*p* < 0.05). Under 40% 1-RM condition, ECC mean velocity was equal with CON mean velocity at the initial part and higher than CON mean velocity at the last part (*p* < 0.01).

**Figure 2:**
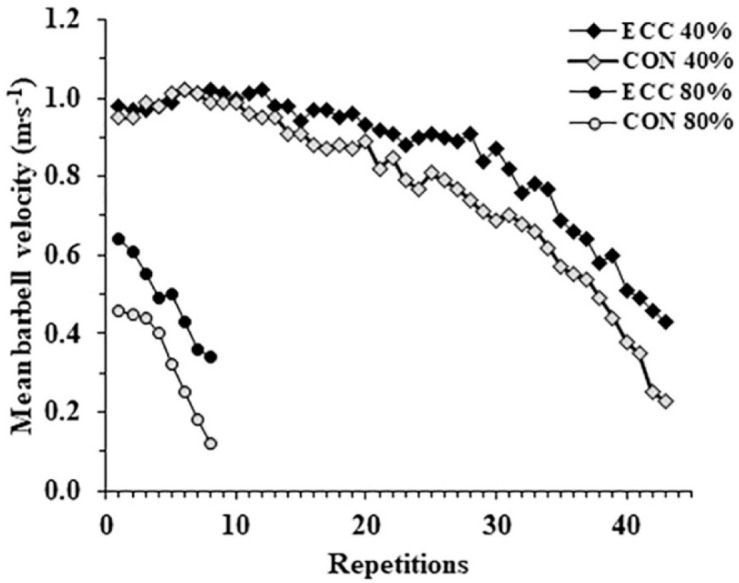
Mean concentric (CON) and eccentric (ECC) barbell velocities per repetition during the two conditions of a representative participant.

Figure 2 shows the decline in mean concentric and eccentric barbell velocities during the sets performed to failure against two different loads for a representative participant. Two-way ANOVA for velocity loss showed a significant interaction (*p* = 0.022 η^2^ = 0.46) and post-hoc tests showed that velocity loss was lower in the ECC compared with the CON phase for both loads (40%1-RM: ECC: −36 ± 21% vs. CON: −63 ± 14%, *p* < 0.001; 80%1-RM: ECC: −26 ± 15% vs. CON: −59 ± 9%, *p* < 0.001).

### sEMG Activity

**Figure 3:**
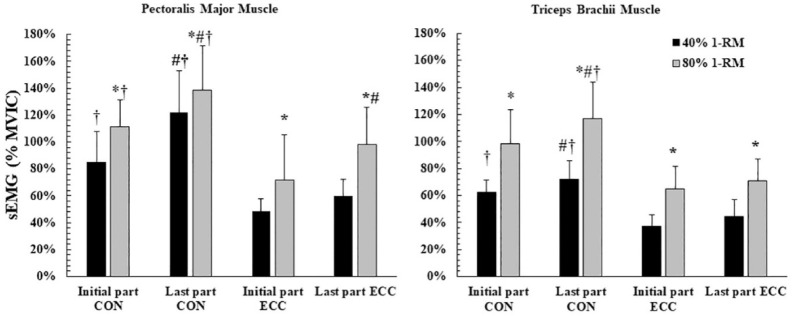
sEMG activity of the pectoralis major (left panel) and triceps brachii muscles (right panel) presented as the percentage of sEMG measured during the maximal voluntary isometric contraction (%MVIC) during the bench press against two different loads. *: *p* < 0.05 from the 40% 1-RM condition; #: *p* < 0.01 from the initial part; †: *p* < 0.01 compared with ECC muscle actions

Three-way ANOVAs revealed significant three-way interactions (load x muscle action x part) for sEMG of the pectoralis major (*p* = 0.012; η^2^ = 0.52) and triceps brachii muscles (*p* = 0.039; η^2^ = 0.39). sEMG activity during both phases (CON and ECC) was higher during the 80% 1-RM than during the 40% 1-RM (*p* < 0.05) for both muscles (Figure 3). During the CON phase, sEMG of the pectoralis major at the last part of the set (fatigue) increased by 48.6% under the 40% 1-RM condition (from 85 ± 23 to 122 ± 31 %MVIC; *p* < 0.001) and by 24.8% under the 80% 1-RM condition (from 111 ± 20 to 138 ± 33 %MVIC; *p* = 0.001). Similarly, sEMG of the triceps brachii during the CON phase increased under the 40% 1-RM condition by 15.7% (from 62 ± 9%MVIC to 72 ± 14%MVIC; *p* = 0.020) and by 21.0% under the 80%1-RM condition (from 98 ± 25 %MVIC to 117 ± 27 %MVIC; *p* < 0.001). During the ECC phase, there was no change in sEMG in the last part of the set for both muscles and both loads (*p* = 0.262 to 0.107) with the exception of the sEMG of the pectoralis major muscle under the 80% 1-RM condition (Figure 3). sEMG was higher during the CON compared with the ECC phase in both muscles (*p* < 0.01).

## Discussion

This is the first study comparing sEMG responses and changes in barbell velocity during the ECC versus the CON phase of the movement in the bench press exercise performed with maximum velocity to failure, against low (40% 1-RM) and high loads (80% 1-RM). One main finding was that barbell velocity loss was less in the ECC than the CON phase of the movement for both loads, and thus fatigue was greater in the CON compared with the ECC phase. In addition, sEMG in the ECC phase was lower than in the CON phase for both muscles, while sEMG during the ECC phase did not increase at the end of the set except for 80%1-RM in the pectoralis major muscle. sEMG during the CON phase was greater at the end of the set in both muscles and loads. Thus, fatigue and sEMG were lower during the ECC compared with the CON phase of the movement. Also, ECC mean barbell velocity was higher compared with CON mean velocity in both loads and parts of the set except the initial part under the 40% 1-RM condition.

Previous studies (Sakamoto et al., 2012; Tsoukos et al., 2021b) have shown that during the course of fatiguing resistance exercise, barbell velocity (mean and peak) decreases and sEMG activity increases, as also found in the present study. One of our previous studies (Tsoukos et al., 2021b) examined changes in barbell velocity and sEMG activity of prime movers with three different loads in the bench press exercise and found that for all loads, sEMG activity was greater in the last part of the set compared to the initial part (Tsoukos et al., 2021b). This was explained by increased neural drive and/or by the additional recruitment of motor units in order to compensate for reduced force production (Aagaard et al., 2002; Del Vecchio et al., 2019; Henneman and Olson, 1965; Moritani et al., 1982). Another finding was that the maximum rate of the sEMG increase (ΔEMG/Δtime) (Tsoukos et al., 2021b), which is a measure of muscle activation, was higher in the last part of the set, possibly indicating an increase in neural drive (Aagaard et al., 2002).

In the present study, velocity loss was higher in the CON compared with the ECC phase of the movement. One possible explanation may be that the relative load, i.e., the load expressed as a percentage of maximum strength, was lower in the ECC than in the CON phase, since ECC muscle strength is higher than CON strength (Kelly et al., 2015). A previous study compared maximum strength and repetitions to failure during isolated ECC and CON bench presses and found that ECC maximum strength (1-RM) was 124% of CON 1-RM, while the number of repetitions completed at 90% 1-RM was significantly greater under the ECC compared with the CON condition (7.8 vs. 4.4 repetitions) (Kelly et al., 2015). Assuming that in the present study ECC 1-RM was 124% of the CON 1-RM, then the load during the ECC phase of the bench press was calculated to be around 31% and 65% of the maximum eccentric 1-RM, while the relative load in the CON phase was 40% and 80% of the CON 1-RM.

Another possible cause for greater fatigue in the CON phase may be the higher energy requirement of CON compared with ECC muscle actions (Clarkson and Newham, 1995; Herzog, 2018). Research has shown that ECC muscle actions have significantly lower energy requirements or metabolic cost compared with CON muscle actions (Clarkson and Newham, 1995; Herzog, 2018) and as a result, CON muscle actions induce greater fatigue (Gonzalez-Izal et al., 2014; Pasquet et al., 2000; Potvin, 1997). Gonzalez-Izal et al. (2014) compared ECC and CON muscle actions and found the CON protocol resulted in greater muscle force losses, blood lactate concentrations, and changes in sEMG variables. Similarly, Potvin (1997) showed that at the fatigue state, sEMG increased by 35% in CON and only by 10% in ECC contractions, and Westing et al. (1991) reported that despite that ECC torque was 20% to 146% higher than CON torque, sEMG activity was 7 to 31% lower under eccentric loading than velocity-matched concentric loading. Therefore, the result that ECC velocity in the present study was maintained during fatigue and sEMG in ECC muscle actions was not changed from the initial to the last repetitions of the set may be attributed to the lower relative load and the lower energy cost of muscle actions during the ECC than the CON phase.

Another finding of the present study was that ECC mean velocity was significantly higher compared with CON mean velocity in both loads and parts of the set except the initial part under the 40% 1-RM condition. This is in agreement with previous studies where participants performed the exercise with the intention to move as fast as possible (Sampson et al., 2014). For example, during a fast elbow flexion/extension completed against a load of 6RM performed to repetition failure, mean ECC and CON velocities were 0.57 ± 0.03 m·s^−1^ vs. 0.43 ± 0.02 m·s^−1^, respectively (Sampson et al., 2014). The faster ECC phase during the bench press exercise may be due to the influence of gravity which assisted the downward ECC movement, as the instruction to participants was to move the barbell upward and downward as fast as possible from the start of the set until failure. The equal ECC and CON velocity in the initial part of the 40% 1-RM condition may be explained by the very fast movement (i.e., barbell velocity was almost two-fold faster compared with 80% 1-RM), while the influence of gravity was less.

The finding that sEMG was greater during the 80% 1-RM condition compared with the 40% 1-RM in all parts and muscle actions is readily explained by the large difference between the two loads. Previous research has shown that there is a non-linear increase in sEMG activity with progressing loads (Vigotsky et al., 2018). For example, sEMG activity of the pectoralis major and triceps brachii muscles during the bench press exercise to instant exhaustion was lower when a load of 40% 1-RM was used, compared with the loads of 60% and 80% 1-RM (Tsoukos et al., 2021b). Thus, it may be hypothesized that fewer motor units were activated under the 40% 1-RM condition, as a result of lower neural drive to the muscles (Aagaard et al., 2002; Del Vecchio et al., 2019; Henneman and Olson, 1965). Another possible explanation for the lower sEMG activity under the low load condition (40 vs. 80% 1-RM) may be the longer deceleration phase during each repetition when the load was lighter. Research has shown that when lifting light loads, the acceleration phase ends earlier and peak velocity is achieved sooner, resulting in a longer deceleration phase compared with heavy loads (Michael Frost et al., 2008). According to Newton’s second law of motion, this implies that the participant exerts lower forces on the barbell and thus, it may be reasonable to observe lower sEMG activity (Michael Frost et al., 2008).

It should be noted that when bench pressing against different loads, and as a result at different speeds, the contribution of the involved muscles may be slightly modified (Król et al., 2010). In the present study, a limitation was that sEMG of two main prime movers was examined (pectoralis major and triceps), but sEMG of the anterior deltoid was not monitored due to practical constraints. The anterior deltoid and pectoralis muscles are maximally activated after the “sticking point” or “sticking period”, which is the phase where the speed of the barbell is minimized (van den Tillaar and Ettema, 2009, 2010). This switch of activation from the maximum activity of the triceps to the maximum activity of the pectoralis and the anterior deltoid muscles is more pronounced during fatigue (van den Tillaar and Saeterbakken, 2014), and in our study it was evidenced as an increase in sEMG activity of the pectoralis major muscle. The results of the present study should be interpreted with caution as the performance level of male participants was moderate (1-RM bench press: 99.0 ± 15.6 kg, relative strength: 1.26 ± 0.18 kg•kg^−1^ body mass) and may not be generalizable to experts who present different kinetic and kinematic characteristics during this exercise (Mausehund and Krosshaug, 2023).

## Conclusions

The present study showed that fatigue, as measured by velocity loss, was lower during the ECC than the CON phase of the bench press movement when the exercise was performed with maximum velocity to failure, irrespective of the load (40% or 80% of 1-RM). Also, sEMG was lower in the ECC than the CON phase for all loads, and increased at the end of the set only during the CON phase, while it remained unchanged in the ECC phase, except for the pectoralis major when the heavier load was applied. These findings confirm that in the bench press exercise, the ECC muscle action is less prone to fatigue although the ECC mean velocity is higher. These differences in fatigue during the CON and ECC phases of the bench press exercise using free weights, should be considered when designing resistance training protocols for sports requiring upper body strength. For example, to maximize the training stimulus, strength and conditioning coaches may use eccentric overload methods (i.e., use a higher load during the ECC phase) to take advantage of the differences in fatigue between the ECC and CON phases of the movement, or they may require the athlete to continue with ECC only movements when CON fatigue is reached.
